# The Navy Medical Service

**Published:** 1911-09-16

**Authors:** 


### The Hospital

A Journal of

The Medical Sciences and Hospital Administration.

New Series. No. 242, Vol. IX. [No. 1314, Vol. L.] SATURDAY, SEPTEMBER 16, 1911.

### The Navy Medical Service.

In the last issue of The Hospital reference was made (p. 595) to the post-graduate aspects of the new regulations which have been issued by the Admiralty, effecting various important alterations in the medical branch of the Royal Navy. There are, however, many other matters dealt with in the regulations and in the Admiralty circular letter which accompanies them; and in certain respects some of these changes are perhaps as likely to be as far-reaching in their effects as are those concerning graduate study. A careful consideration of the subject renders it tolerably clear that there is still room for further improvement in the conditions of the service; and we are pleased to see in the *Morning Post* a strong hint that further modifications of them are in view. One thing about the navy medical service is undeniable, and that is that there has long ceased to be any great competition to enter it. As a matter of fact, the personnel of the service is wonderfully good, all things considered; but it is not a healthy state of affairs when competition for entrance is reduced almost to a formality. Another matter which shows that the conditions have not been attractive enough is that for some years the full establishment has never been completed and that the department has been in a chronic state of working below full strength. As a proof that this service still has unremedied grievances, the latter of Messrs. H. C. and E. H. Ross in the *Morning Post* of September 12 should be carefully studied.

To deal first with pay and allowances—the sinews of naval no less than of military warfare—it seems that the rates of minimum and maximum pay of every grade remain substantially the same as now; but increases will be granted biennially instead of every four years, each increase being of half the amount of the increase previously allowed. In practice this concession is by no means negligible, and a little consideration will show that its effect is practically the same as would be that of bringing each of the increases on the old system one year nearer to the members of the service. The alterations in the complicated and inconsistent details of "charge pay" are also advantages, though they affect but a small proportion of those actually serving. The details of the post-graduate scheme show that this question is one which the Admiralty are in earnest over, and that in future there will be more chance than in the past for the energetic and well-equipped surgeon to obtain recognition and advancement, rather than a dead level of promotion by strict seniority, irrespective of merit. During the fifth, sixth, or seventh year of his service every surgeon must attend a six-months' course of post-graduate study and pass an examination at the end of it, failing which he cannot be promoted to the grade of staff surgeon. Moreover, he who obtains eighty-five per cent. of marks in this examination will thus acquire eighteen months' seniority; and he who gets seventy-five per cent. will similarly have twelve months of the same sort of reward. On the other hand, those who twice fail to obtain half marks will be compulsorily retired after eight years' service, and will be able to command only £500 gratuity, instead of the £1,000 allotted to those who retire voluntarily after having passed it (and after eight years' full-pay service). It is thus apparent that ample incentives to diligence in the post-graduate classes are being provided, though they are partly in the nature of punishment for slackness as well as of material advantage for keenness.

In another direction changes are made which are possibly in some degree the upshot of a court-martial which excited much interest and comment in Navy circles not very long ago. Thus the senior medical officer of the flagship of any fleet is now to be a member of the staff of the Commander-in-Chief, and to act as his expert adviser on the hygiene and other medical problems of the fleet or any unit of it. Besides reporting direct to the Admiralty, he will have direct access to the Admiral on all occasions. Individual medical officers are on occasions hampered in their duty by the caprice or prejudice of an executive officer who may have small sympathy with up-to-date medicine, and it is very difficult for such surgeons to carry out what they conceive to be their duty without making enemies of those under whom they serve. In future the principal medical officer of a fleet will be able to bring to bear on such cases an influence derived from his new position which will be much more effective in securing that a zealous and efficient surgeon shall not be overridden by an ignorant captain on questions vitally affecting the health of the ship's company. Another reform which will tend to have the same effect is that which allots to the principal medical officer the duty of making an independent report on the professional conduct and ability of each surgeon in his fleet. Thus there is provided a check on the periodical reports on medical officers from their respective superior officers. It is obviously fairer to surgeons that a professional critic of their own branch of the service shall be allowed a voice in reports which may otherwise emanate from those who have slight sympathy with the surgeon's work. One suggestion which the Admiralty will do well to ponder carefully is that at least one medical officer should sit as a member of every court-martial held upon an officer of the Navy Medical Service. We cannot conceive any valid objection, and it is easy to see how such a regulation would prove valuable.

Whether these reforms will restore the lost popularity of the service remains to be seen. It is quite possible that the concentration of the fleets in and about home waters has something to do with the falling off, as thereby the attractions of foreign travel and sport have been much curtailed for officers of this service when afloat.

